# Effects of Vertical Water Mass Segregation on Bacterial Community Structure in the Beaufort Sea

**DOI:** 10.3390/microorganisms7100385

**Published:** 2019-09-24

**Authors:** Yunyun Fu, Richard B. Rivkin, Andrew S. Lang

**Affiliations:** 1 Department of Biology, Memorial University of Newfoundland, 232 Elizabeth Ave., St. John’s, NL A1B 3X9, Canada; yunyun.fu@mun.ca; 2 Department of Ocean Sciences, Memorial University of Newfoundland, 0 Marine Lab Rd., St. John’s, NL A1C 5S7, Canada; rrivkin@mun.ca

**Keywords:** Arctic Ocean, Alphaproteobacteria, Rhodobacterales, Pelagibacterales, stratification, ice cover, depth

## Abstract

The Arctic Ocean is one of the least well-studied marine microbial ecosystems. Its low-temperature and low-salinity conditions are expected to result in distinct bacterial communities, in comparison to lower latitude oceans. However, this is an ocean currently in flux, with climate change exerting pronounced effects on sea-ice coverage and freshwater inputs. How such changes will affect this ecosystem are poorly constrained. In this study, we characterized the bacterial community compositions at different depths in both coastal, freshwater-influenced, and pelagic, sea-ice-covered locations in the Beaufort Sea in the western Canadian Arctic Ocean. The environmental factors controlling the bacterial community composition and diversity were investigated. Alphaproteobacteria dominated the bacterial communities in samples from all depths and stations. The Pelagibacterales and Rhodobacterales groups were the predominant taxonomic representatives within the Alphaproteobacteria. Bacterial communities in coastal and offshore samples differed significantly, and vertical water mass segregation was the controlling factor of community composition among the offshore samples, regardless of the taxonomic level considered. These data provide an important baseline view of the bacterial community in this ocean system that will be of value for future studies investigating possible changes in the Arctic Ocean in response to global change and/or anthropogenic disturbance.

## 1. Introduction

Bacterioplankton are the most abundant organisms in the sea, where they exist in complex and diverse communities. They contribute to the cycling, remineralization and transformation of material and energy in marine ecosystems and channel carbon and essential nutrients to higher trophic levels [1,2,3]. However, due to the complexity in both microbial community dynamics and ocean ecosystems, the determinants of microbial diversity, distribution, and community composition remain poorly understood in marine environments.

Previous research has shown that diverse biotic and abiotic factors affect the activity and structure of microbial communities, and these include temperature [4], depth, stratification and mixing in the water column [5,6,7,8,9], light availability [10], latitude [4,11], nutrient availability [12,13], water mass [14,15,16,17,18], inter-species interactions and competition, and predation by viruses and protists [19,20,21]. It is also important to note that there are connections and overlap among these different factors.

Over the past few decades, several microbial groups have become foci of research in marine microbiology, due to their abundance and widespread distributions. The widespread and abundant groups include members of the alphaproteobacterial orders Pelagibacterales (i.e., SAR11) [6,22,23,24,25] and Rhodobacterales (i.e., roseobacters) [26,27,28,29,30]. These two lineages are found primarily in the upper ocean and appear to use distinct strategies for their success in marine environments. The Pelagibacterales are characterized by very streamlined genomes and it was a challenge to bring a representative into laboratory culture [10], whereas roseobacters tend to have many genes for diverse metabolic capacities and many representatives are readily cultured in the lab [31]. The spatial and temporal distributions of these two groups have been studied in temperate and tropical oceans [9,32,33,34,35,36,37,38], but less so in polar oceans. However, previous studies have documented they are also found in high abundance in the Arctic Ocean [39,40,41,42].

Polar oceans have distinct physical features, and therefore distinct bacterioplankton communities, compared to the tropical and temperate oceans. For example, the Arctic Ocean is characterized by low temperatures, salinities, underwater irradiances and primary production, complex hydrodynamics, extensive ice coverage and inorganic nutrient concentrations, and generally low bacterial production [43]. The Arctic Ocean receives approximately 10% of the global freshwater input [44], leading to large spatial gradients in physicochemical conditions, such as temperature, salinity, and biogeochemical properties [45]. These gradients have significant effects on the microbial communities in coastal Arctic waters. In particular, large amounts of sediments are associated with the freshwater input, estimated at 124 million tons annually [46,47], and sediments also harbor unique microbial communities [48]. Previous studies have reported significant differences in microbial abundances and rates of production along river-estuary-sea transitions [48,49]. The presence of sea ice also is a significant factor in the Arctic Ocean. It limits light penetration into the water column and prevents mixing of the surface waters. During melting, microbes present in the sea ice are released into the surrounding water and the freshwater lowers the salinity, enhancing water column stratification.

Marine bacterioplankton have a profound influence on nutrient cycling in the ocean, including the transformation among particulate and dissolved organic and inorganic carbon pools. Temperature, substrate concentrations and bacterioplankton community structure are considered to be the major factors controlling the rates and patterns of carbon transformation [50,51], which therefore have important implications with respect to climate change. In regions that are highly sensitive to climate impacts, such as the polar areas [52,53], it is important to study both bacterioplankton processes as well as the community structure, with particular emphasis on the groups that potentially have biogeochemical significance. From studies in temperate and tropical oceans, members of the Pelagibacterales and Rhodobacterales are implicated in many ecosystem functions that affect carbon cycling, remineralization and sequestration.

In this study, we characterized the overall bacterioplankton community structure, with particular emphasis on these two lineages, in the Beaufort Sea in 2009 at stations spanning from the Mackenzie River plume on the continental shelf to the ice-covered offshore. The susceptibility of this region to potential impacts from both climate change and anthropogenic disturbance makes these data extremely relevant for potential future assessments of such factors.

## 2. Materials and Methods

### 2.1. Sample Sites, Physicochemical Parameters, and Biological Properties

**Figure 1:**
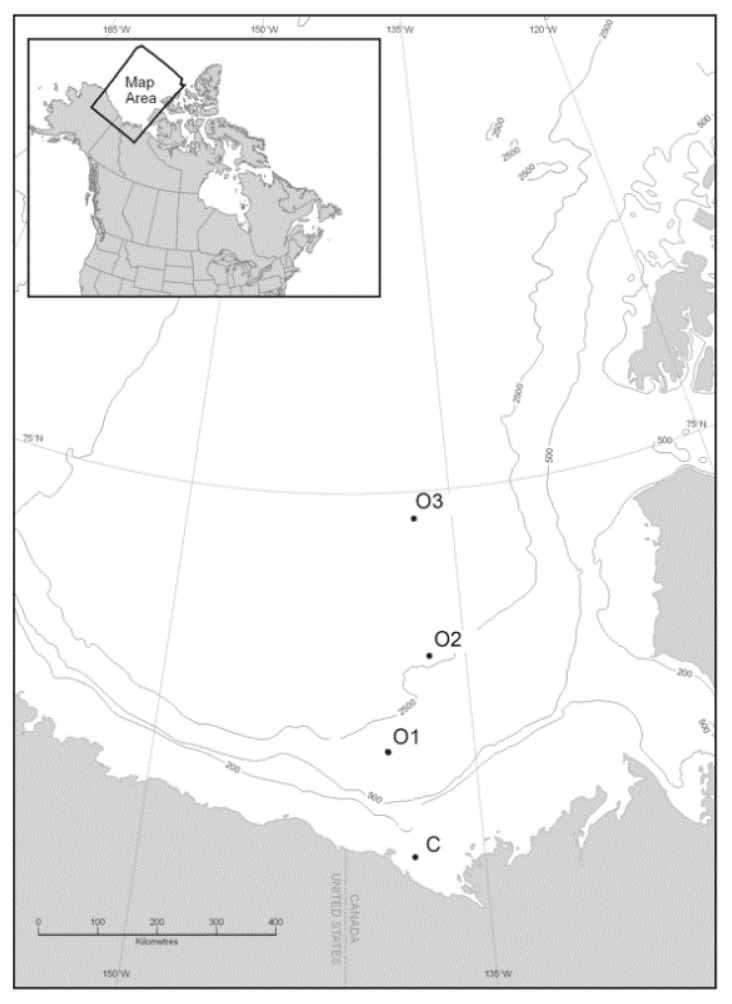
Sampling sites in the Beaufort Sea. C, coastal; O, offshore.

Water samples were collected during the Canadian IPY-GEOTRACES program (27 August 2009 through 12 September 2009) aboard the CCGS Amundsen on a transect from the mouth of the Mackenzie River to the offshore basin of the Beaufort Sea (Figure 1, Table 1), with one station in coastal Beaufort Sea (C) and three stations offshore: upper slope (O1), lower slope (O2), and basin (O3). Water samples were collected using a rosette sampler equipped with a Seabird 911*plus* Conductivity-Temperature-Depth (CTD) probe, an in situ fluorometer (SeaPoint 2465), an irradiance sensor (QCP-2300, Biospherical Instruments), and 12-L Go-Flo Niskin bottles. Physicochemical, biological and hydrographical parameters were measured at all stations and included depth, temperature, salinity, sea ice coverage, chlorophyll *a* (Chl *a*) concentration, nitrate concentration, nitrite concentration, and phosphate concentration. The depths of sample collection were based on the vertical profiles of water structure (i.e., density, temperature and salinity) and downwelling irradiance at each site in order to sample distinct water masses and biological characteristics. As possible, samples were collected at the surface and/or the depths with 50%, 1%, and/or 0.1% light intensity compared to the surface (Table 1).

Water mass segregation was determined based on the measured water temperature, salinity and depth [54]. A principal component analysis (PCA) of water temperature, salinity and depth for each sample was performed to verify the water mass segregation. A PCA of physical parameters and Chl *a*, N, and P concentrations was conducted to further investigate the water properties of these samples. Data for N and P concentrations were not available for sample O2–10 so it was excluded from this PCA.

Bacterial abundance (BA) was determined by flow cytometry, using a FACSort™ (Becton Dickinson) equipped with a 488-nm argon-ion laser, and Acridine Orange direct counts. Samples for flow cytometry were fixed in a final concentration of 1% paraformaldehyde for 10 min at room temperature, rapidly frozen in liquid nitrogen within one hour of sample collection and stored at −80 °C until analysis. Samples were stained with SYBR Green 1 and analyzed using standard protocols [55,56]. Flow cytometry counts were verified by comparison to Acridine Orange direct counts [57]. Bacterial production (BP) was calculated from [^14^C]-leucine (final concentration of 10 nM leucine) incorporation during 6-h dark incubations at ambient temperatures and using a standard conversation factor of 3.1 kg C mol^−1^ [^14^C]-leucine incorporated [58]. Bacterial biomass (BB) was estimated from bacterial abundance and cellular carbon content, computed from measured cell sizes using the geometric mean cell volume for each sample [59]. The bacterial growth rate (BGR) was calculated assuming exponential growth of bacteria during the bacterial production incubations: *SGR* = *log*_e_ [(BP_Δ_*t* + BB)/BB]/Δ*t*, where BP_Δ*t*_ is bacterial production obtained during the incubation, and Δ*t* is the incubation time in days.

### 2.2. DNA Extraction, PCR Amplification and Sequencing

Water (500 mL) was filtered through 0.22-µm polycarbonate filters (Millipore), which were then stored frozen until DNA extraction in 2013. Total DNA was extracted from individual filters as described previously [18]. Briefly, one half of each filter was incubated in 2 mL lysis buffer (10 mM Tris-HCl, 1 mM EDTA, 0.8 M sucrose; pH 8) containing 1 μg mL^−1^ lysozyme for 30 min at 37 °C. This was followed by addition of 200 μL of 10% (w/v) cetyltrimethylammonium bromide (CTAB) in 1.4 M NaCl and incubation for 30 min at 65 °C. Samples were then sequentially extracted with phenol-chloroform-isoamyl alcohol (25:24:1) and chloroform-isoamyl alcohol (24:1) and DNA was precipitated from the aqueous phase by addition of an equal volume isopropanol. After centrifugation, the DNA pellets were washed with 70% ethanol, dried, and dissolved in 200 μL TE buffer (10 mM Tris-HCl, 1 mM EDTA; pH 8).

The 16S rRNA gene V6 region was amplified using previously published primers [60], with the addition of barcode and sequencing adaptor sequences. The barcodes and adaptors were designed to allow sequencing using the Ion Torrent Personal Genome Machine (PGM) (Thermo Fisher Scientific) and each of the 11 samples was subjected to amplification with one of 11 different barcoded primer sets.

The target gene fragments were amplified by PCR in 50-µL volumes containing 0.375 µM each of the forward and reverse primers, 800 µM dNTPs (NEB), 0.5 U Phusion Hot Start polymerase (Thermo Fisher Scientific), and 1X Phusion HF Buffer. Thermocycling conditions were: 30 s at 98 °C followed by 30 cycles of 98 °C for 10 s and 72 °C for 30 s, and a final incubation at 72 °C for 2 min. The PCR products were purified using Agencourt AMPure XP reagent (Beckman Coulter) using 1.4X volume of the AMPure XP reagent and quantified using the Bioanalyzer High Sensitivity DNA Analysis kit (Agilent). The amplicons were then each diluted to 26 pmol μL^−1^ and pooled together in equal volumes. The mixed amplicons were ligated to Ion Sphere Particles (ISPs) and amplified on the OneTouch2 instrument using the 200 bp kit (Thermo Fisher Scientific), and the sequencing was performed on a PGM sequencer using the Ion PGM 200 bp v2 sequencing kit (Thermo Fisher Scientific).

### 2.3. Sequence Analyses

The raw sequence data were processed and analyzed using mothur [61]. The sequence reads were binned according to barcode and underwent strict quality filtering. Any read containing an error in the barcode, primer, or adaptor regions was removed from the data set. Reads with an average quality score <25 were also removed.

The remaining sequences were aligned with the SILVA v.119 16S rRNA gene database via mothur (http://www.mothur.org/wiki/Silva_reference_files) to obtain the phylogenetic and taxonomic profiles of each community. Each sequence was assigned a taxonomic label according to the database, with the assumption that the proportional representations of sequences in the data represent the proportional representation of the taxa in the samples or are at least comparable among our samples, which were all processed in parallel and using the same methods. Taxa with total sequence counts lower than 1% of the total community were removed from the analyses. The sequences were grouped together into operational taxonomic units (OTUs) at 97% sequence identity. Phylogenetic analyses were conducted with these assigned OTUs.

Due to a lack of resolution within Pelagibacterales in the SILVA reference file, the sequences that were assigned to this group were subjected to an additional analysis with reference sequences from subclades identified [62] independently. The Pelagibacterales sequences were grouped into OTUs at 97% nucleotide identity and a representative sequence from each OTU was selected using mothur. The reference sequences from the SAR11 subclades [6,22,63,64,65] were obtained from the NCBI database (Table S1). The reference sequences were trimmed to the corresponding V6 region available for our data and aligned with the Pelagibacterales OTU representatives in MEGA6 [66]. Pairwise distances were calculated to determine the relationships of our OTUs with the SAR11 subclade reference sequences. A phylogenetic tree was constructed in MEGA from the alignment using the neighbor-joining method with 1000 bootstraps to evaluate the relationships of the Pelagibacterales OTUs and the defined SAR11 subclades.

The taxonomic diversity of each sample was evaluated using the Shannon index [67] and the inverse Simpson index [68,69]. Community richness was assessed by the Chao-1 richness estimate [70]. Similarities among samples were evaluated using Yue and Clayton measure of dissimilarity (θ_yc_) [71]. These analyses were all performed in mothur.

### 2.4. Statistical Analyses of Bacterial Community Structure

Principal component analysis, performed in R-package vegan2.2–0 [72], was used to investigate the relationships among the bacterial communities and to assess the impact of various environmental parameters on these relationships. The PCA was performed based on the proportional amounts of the different taxonomic groups in the samples at four different taxonomic levels: phylum, major classes in the phylum Proteobacteria, major genera and lineages in the order Rhodobacterales, and OTUs within the Pelagibacterales. The proportional amount of each taxonomic unit in a sample was considered as the proportion of the 16S V6 sequences assigned to that unit [17,48,73]. The correlation between each environmental parameter and the PCA ordinates were calculated, using the function envfit in vegan2.2–0, to show which parameters contributed to the separation of the bacterial communities. Those parameters showing significant correlations (*p*-value < 0.05) were reported on the plots, and the correlation coordinates and all *p*-values are reported in Table S2.

The effects of environmental factors (station, water mass, temperature, salinity, and density) and biological factors (BB, BA, BP, BGR, and Chl *a*) on diversity were estimated using Pearson correlations or analysis of variance (ANOVA) tests. Bacterial diversities were evaluated for the total bacterial community and the groups Rhodobacterales and Pelagibacterales. The categorical factors (station, water mass) were used in ANOVA tests and the numerical factors used for Pearson correlations.

## 3. Results

### 3.1. General Station and Sample Characteristics

The sampling station locations are shown in Figure 1 and the characteristics for each sample are in Table 1. Water sample temperatures ranged from −1.41 °C to 3.13 °C, with higher temperatures at the coastal station and lower temperatures at the offshore stations. The coastal station (C), located on the continental shelf, had no sea ice coverage. Sea ice increased away from the coast and reached 90% coverage at the furthest offshore station (O3) (Table 1). The water column in the southern Beaufort Sea is vertically stratified and composed of waters originating from both the Pacific and Atlantic. Based on a previous detailed study on water masses in this area [54], we assigned the 9 samples from the offshore stations into three categories: upper polar mixed layer (UPML), lower polar mixed layer (LPML) and Pacific summer water (PSW). The two samples from station C are influenced by the Mackenzie River discharge. Their water temperature, salinity and other chemical properties deviated from the offshore stations at similar depths. As a result, the two samples from station C were assigned to a separate water mass (Table 1).

**Figure 2:**
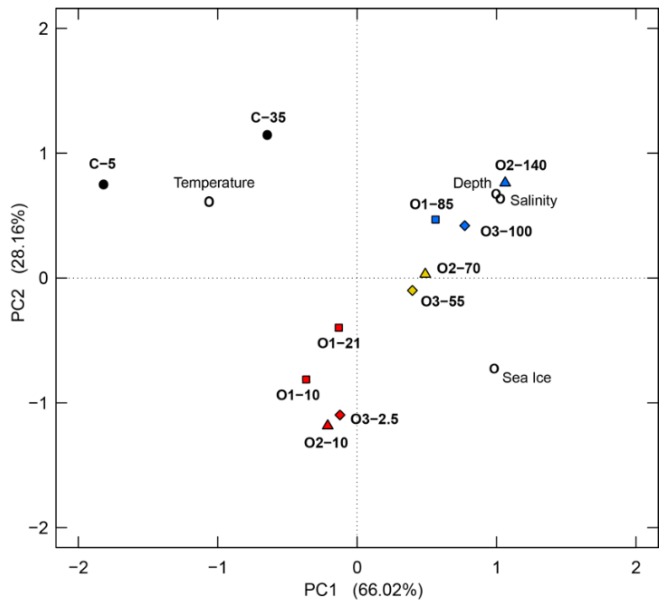
Principal component analysis (PCA) based on the environmental properties of the 11 samples. The open circles represent the environmental properties and the closed symbols represent the samples. The samples are colored according to water mass: red, UPML; yellow, LPML; blue, PSW; black, coastal. The shapes represent different stations: C, circles; O1, squares; O2, triangles; O3, diamonds.

Principal component analysis was performed based on the physical environmental parameters (temperature, salinity, depth, and sea ice converge) (Figure 2). This analysis supported the water mass segregation described above as did a further PCA test with both the physical and chemical parameters, although with a greater separation of the two coastal samples (Figure S1, Table S3).

Bacterial abundance (BA) ranged from 8.8 to 53 × 10^4^ cells mL^−1^, with the highest values at the coastal site and a general decreasing trend by depth in the offshore samples (Table 2). The trends for BB, BP and BGR were also similar to what was found for BA (Table 2). The Chl *a* differed among samples and sites, with the highest concentrations again at the coastal station, and the highest Chl *a* concentrations at the offshore stations were found at the intermediate depths (Table 2).

### 3.2. Bacterial Community Characterizations

A total of 2,213,429 sequences were obtained and 1,613,944 passed quality filtering (Table S4). The number of sequences obtained for the different samples ranged from 48,356 to 205,484 (Table 3), and the estimated OTU richness ranged from 1200 to 4501. The samples with the highest sequence counts did not have the highest taxa richness (Table 3) and diversity estimates were not correlated with the numbers of sequences obtained from the samples (Figure S2).

**Figure 3:**
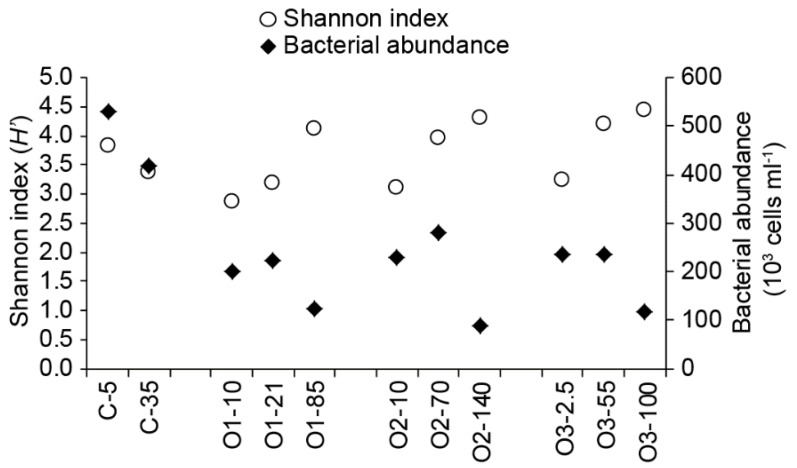
Comparison of the total bacterial community diversity, represented by the Shannon index, and bacterial abundance for the samples in this study. The Shannon index was calculated with OTUs defined at 97% identity.

The Shannon index was used to evaluate the relative total community diversity for all samples, and values ranged from 2.87 to 4.45. For samples from offshore stations, diversity increased with depth, while at the coastal station, the surface sample was more diverse (Table 3). Diversity was highest in the deep offshore samples, where the lowest bacterial abundances were found (Figure 3). The inverse Simpson diversity index (1/D) was also calculated, and values ranged from 5.668 to 14.003 (Table 3). The relationship of depth to this index was the same as for Shannon at each station, with one exception. At station O2, the highest diversity according to the Shannon index appeared at 140 m, whereas the highest diversity according to the inverse Simpson index appeared at 70 m, although the differences between these two samples at this station were fairly small.

### 3.3. Patterns for the Alphaproteobacteria, Rhodobacterales, and Pelagibacterales

**Figure 4:**
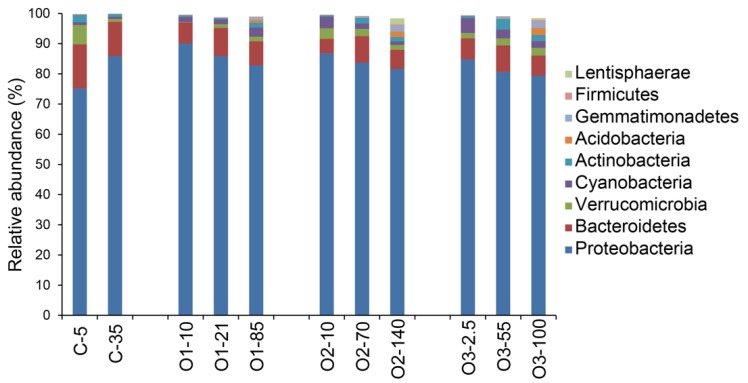
Community composition by bacterial phyla. The samples are indicated on the x-axis by sampling station (see Figure 1) and depth (m).

**Figure 5:**
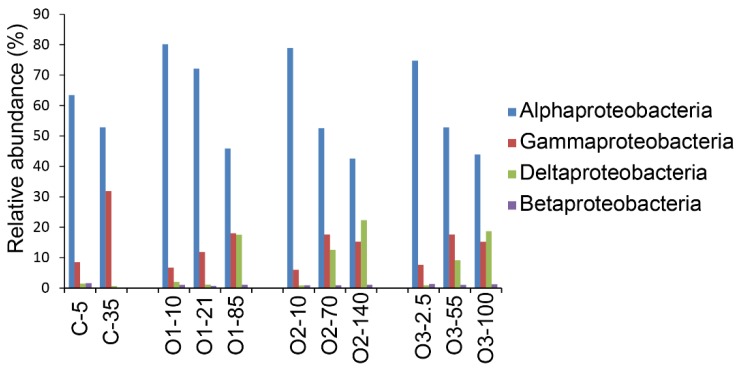
Proteobacterial sequence representation within the bacterial communities. The proportions of sequences from each class are calculated with respect to the total number of sequences obtained for each sample.

Sequences were assigned to taxa based on comparisons with known sequences and the proportions of sequences for the different taxa compared among the samples. Sequences mapping to the phylum Proteobacteria accounted for >70% of the total sequences in all samples (Figure 4). Sequences from Bacteroidetes represented the second most abundant phylum, accounting for 11–15% at the coastal site and approximately 8% in the offshore samples (Figure 4). Within the Proteobacteria, the class Alphaproteobacteria dominated and represented 40–80% of the total sequences (Figure 5). There was a noticeable decrease in the relative abundance of sequences from Alphaproteobacteria with depth (Figure 5).

**Figure 6:**
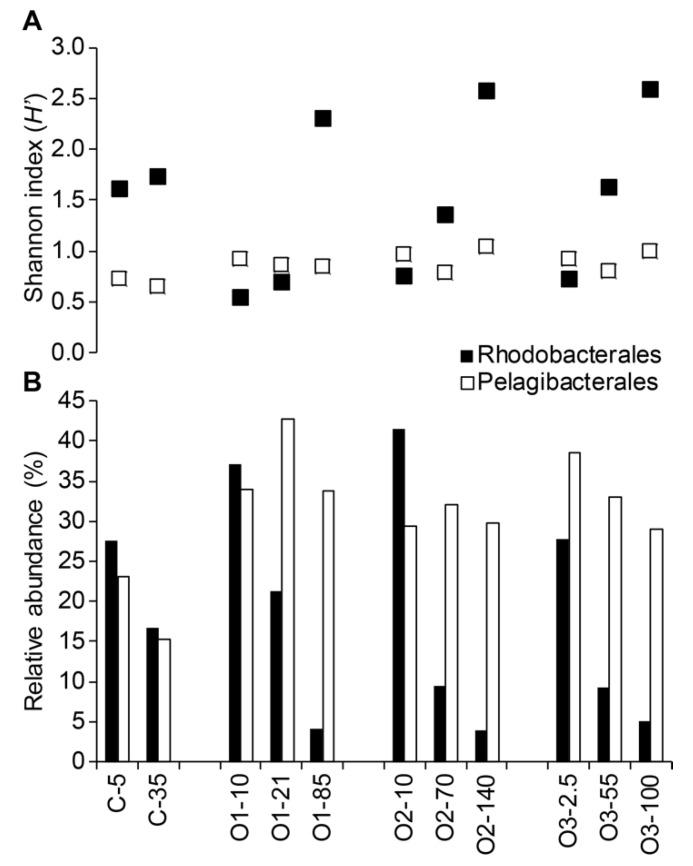
Diversity and relative abundance of sequences from the orders Rhodobacterales and Pelagibacterales. **A**. The diversity, as measured by the Shannon index, within each group for each sample. **B**. The proportion of sequences from each group within the total sequences for each sample.

Below the class level, the Rhodobacterales and Pelagibacterales within the Alphaproteobacteria were the two most dominant taxonomic groups in all samples with the exception of C-35 where the order Rhizobiales represented 19% of all sequences in comparison to 16.6% for Rhodobacterales and 15.2% for Pelagibacterales. The relative proportion of Pelagibacterales was uniformly high in the offshore samples but lower at the coastal site (Figure 6). The highest proportions of sequences from the Rhodobacterales were always found at the surface, where they accounted for 28–41% of the sequences, but they decreased with depth and this was most extreme offshore where their relative abundance dropped to <5% in the deep samples (Figure 6).

Shannon and inversed Simpson diversity indices were also calculated for the Rhodobacterales and Pelagibacterales fractions of the communities in each sample. For the Rhodobacterales communities, Shannon diversity ranged from 0.54 to 2.57 and inversed Simpson diversity ranged from 1.220 to 6.329 (Table 3, Figure 6). Both diversity estimates showed the same pattern of increasing with depth in offshore samples and were highest in the offshore deep samples, corresponding to the PSW water mass (Figure 6), and diversity in the costal samples was intermediate relative to the offshore. For the Pelagibacterales communities, the Shannon indices ranged from 0.65 to 1.04, and inversed Simpson diversity ranged from 1.330 to 1.814 (Table 3, Figure 6). Coastal samples were slightly less diverse compared to the offshore samples. In the offshore samples, there was no obvious pattern of diversity related to depth, but the highest diversity appeared in the PSW layer and the lowest was in the LPML layer.

**Figure 7:**
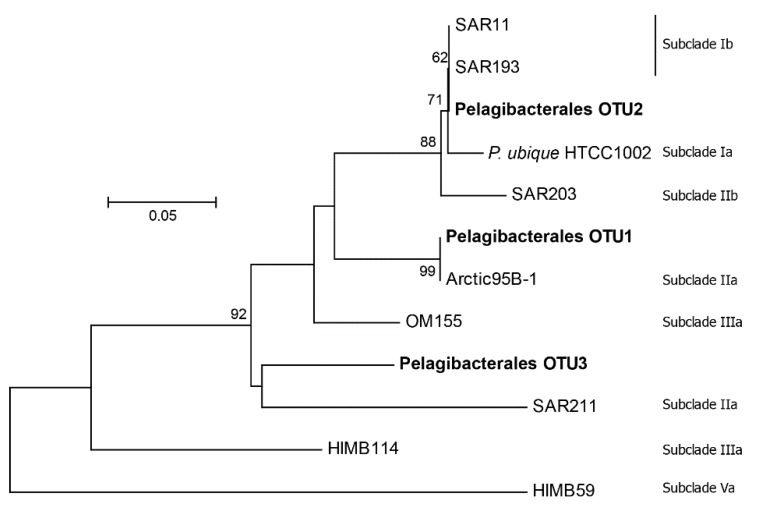
Phylogenetic analysis of Pelagibacterales sequences. The phylogenetic positions of the OTUs identified in our study (OTU1, 2 and 3, in bold) are shown with respect to representative sequences (with original sequence designations) from SAR11 ecotype subgroups (labelled on the right). The tree was constructed with the neighbor-joining method and 1000 bootstrap replicates, with bootstrap values >60 shown at the nodes. The scale bar indicates substitutions per site. Details for the reference sequences are provided in Table S1.

Within the Pelagibacterales the sequences grouped into 3 OTUs (named OTU1, 2 and 3, from most to least abundant). OTU1 was identical with the representative sequence from SAR11 subclade IIa (Arctic 95B-1). OTU2 was identical with the representative sequences from SAR11 subclade Ib (SAR11 and SAR193). OTU3 was more divergent from the defined subclades. It was most closely related to strain OM155, which is a representative from subclade IIIa, at approximately 90% nucleotide identity. This lack of a close relationship with the previously defined subclades was further supported by phylogenetic analysis (Figure 7), suggesting this OTU might represent a novel undefined Pelagibacterales clade.

**Figure 8:**
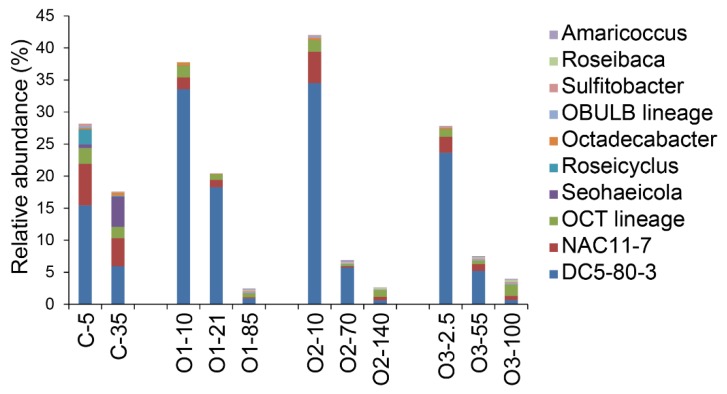
Relative abundance of lineages within the order Rhodobacterales. The values represent the proportions of sequences matching the indicated taxonomic groups within the total number of sequences for each sample.

More detailed examination of the community structure within the order Rhodobacterales showed that most of the sequences were assigned to roseobacter lineages. Sequences assigned to the DC5–80-3 group contributed the largest numbers of sequences in surface and intermediate depth samples (Figure 8). The second most abundant group of sequences was assigned to the NAC11–7 group and these also showed decreasing proportions with depth. The OCT group, which was the third most abundant, did not show a trend with depth (Figure 8).

### 3.4. Bacterial Community Comparisons and the Effects of Environmental Parameters on Community Structure

The effects of physical and biological parameters on the bacterial diversity, estimated by Shannon index, were assessed by analysis of variance (ANOVA) and Pearson correlations (Table 4). Sampling station showed no significant impact on any community. Water mass; however, had statistically significant impacts on diversity for all three community fractions examined. For the other factors, the total community and Rhodobacterales diversities showed the same trends, which were opposite to the Pelagibacterales trend. For the physical parameters, salinity and density were significantly correlated with the diversity in the total bacterial and Rhodobacterales communities, whereas temperature was significantly correlated with the Pelagibacterales diversity. For the biological parameters, no significant correlations were found for the total bacterial or Rhodobacterales communities. However, the Pelagibacterales diversity was significantly negatively correlated with all bacterial biological parameters: BB, BA, BP, and BGR.

**Figure 9:**
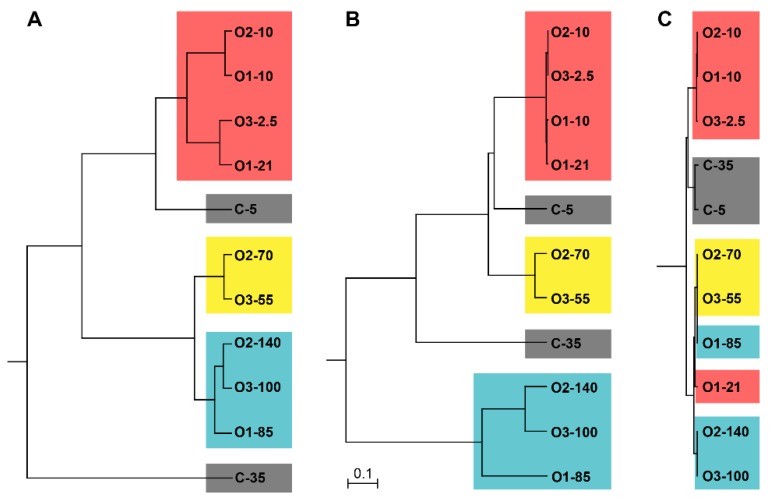
Comparisons of communities at different taxonomic levels across all samples. (**A**) The total bacterial community. (**B**) The order Rhodobacterales. (**C**) The order Pelagibacterales. The comparisons are based on community compositions with 97% identity for definition of OTUs. Communities were compared using the θ_yc_ similarity index. Samples are colored according to water mass: red, UPML; yellow, LPML; blue, PSW; grey, coastal.

The community compositions for the total bacterial, Rhodobacterales, and Pelagibacterales populations were analyzed using θ_yc_ to compare the 11 samples (Figure 9). For the offshore, both the total bacterial (Figure 9A) and Rhodobacterales (Figure 9B) communities were clustered strongly according to water mass, regardless of the station location or physiochemical characteristics. However, the coastal samples did not group together (Figure 9A,B). The Pelagibacterales communities (Figure 9C) showed greater similarity among the samples and the clustering according to offshore water mass was less than for the other two groups, particularly in the LPML and PSW, whereas the coastal samples clustered together.

**Figure 10:**
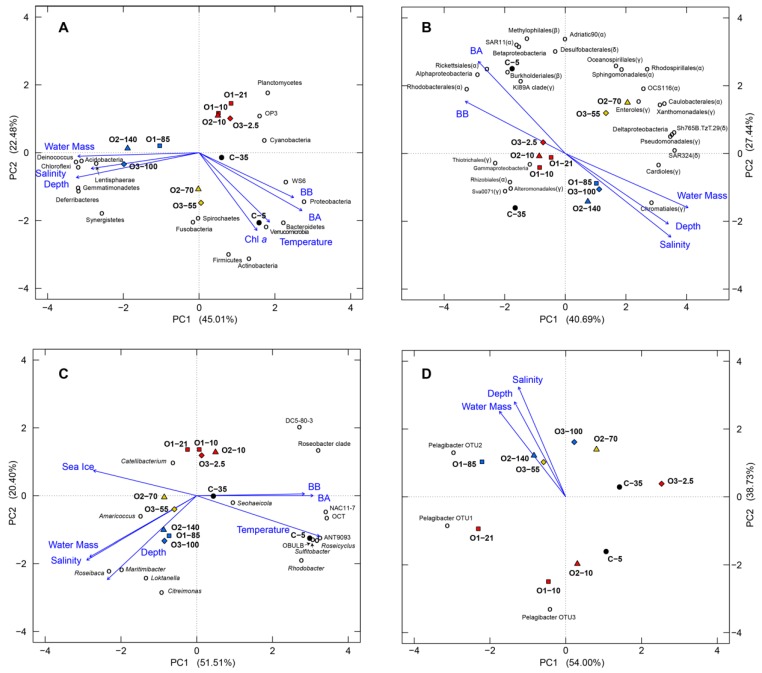
Principal component analysis (PCA) based on the relative proportions of taxonomic groups. Analyses are based on taxonomic levels of phyla (**A**), predominant classes and orders in the phylum Proteobacteria (**B**), major genera and lineages in the order Rhodobacterales (**C**) and the order Pelagibacterales (**D**). The open circles represent the taxonomic groups used for calculating the PCA ordinates and closed symbols represent the 11 samples. The closed symbols are colored according to water mass: red, UPML; yellow, LPML; blue, PSW; black, coastal. The shapes represent the different stations: C, circles; O1, squares; O2, triangles; O3, diamonds. Arrows indicate parameters that were significantly correlated with PC1 and/or PC2 (BA, bacterial abundance; BB, bacterial biomass).

We performed PCA considering four different taxonomic distinctions: phyla, Proteobacteria, Rhodobacterales, and Pelagibacterales (Figure 10). This separated the samples into four clusters. The two coastal (C) samples were always separated from the offshore samples and the offshore samples divided into 3 clusters according to depth: surface, intermediate, and deep. The separation of the depths was clear in all three analyses. BA, BB, salinity and depth showed significant correlations with the first two ordinates in all three PCA plots. The relationship of BA was similar to BB, but opposite to that of salinity and density.

In the PCA based on phyla categorization (Figure 10A), the first and second dimensions (PC1 and PC2, respectively) accounted for 45% and 22.5% of the variation, respectively. The first dimension was significantly positively correlated with BA, BB, Chl *a*, and temperature, but negatively correlated with salinity and depth.

In the PCA based on major classes and orders in the Proteobacteria (Figure 10B), PC1 and PC2 accounted for 40.7% and 27.4% of the variation, respectively. The offshore samples grouped tightly according to water mass, whereas the two coastal samples did not cluster as closely. Bacterial abundance and biomass showed significant negative correlations with PC1, but positive with PC2. Conversely, water mass, depth and salinity showed positive correlations with PC1, but negative with PC2. The different proteobacterial classes (Alpha, Beta, Gamma, and Delta) were distributed widely on the plot (Figure 10B), suggesting their distribution patterns are distinct. The Rhodobacterales showed similar ordination as Alphaproteobacteria, likely due to this order’s high proportion within this class.

In the PCA of major lineages in the Rhodobacterales (Figure 10C), PC1 and PC2 contributed 52.1% and 20.4% of the variation, respectively. Similar to the Proteobacteria communities, the offshore samples formed close groups according to water mass, and the two coastal samples were farther apart. Bacterial abundance and biomass showed very strong positive correlations with PC1, while temperature showed a positive correlation with PC1 and a negative correlation with PC2. Depth, salinity and water mass showed similar effects on the Rhodobacterales communities and were negatively correlated with both PC1 and PC2. Sea-ice-coverage displayed a negative correlation with PC1, and a slightly positive correlation with PC2. The DC5–80-3, NAC11–7, ANT9093, OBULB lineage, OCT group, *Roseobacter* clade, and genera *Loktanella* and *Rhodobacter* contributed strongly to the PC1 ordinate. The DC5–80-3 group and *Roseobacter* clade also showed strong negative relationships with depth.

In the PCA of Pelagibacterales OTUs (Figure 10D), PC1 and PC2 contributed 54.0% and 38.7% of the variation, respectively. Unlike the previous analyses, water mass was less of a defining factor for this taxonomic group and the UPML, LPML and PSW samples were all more widely distributed on the plot. Water mass, salinity, and depth showed strong positive correlations with PC2 and negative correlations with PC1. No biotic parameters showed significant relationships.

## 4. Discussion

The characterization of bacterial communities in the Beaufort Sea suggest that water mass to be the primary determinant of the observed spatial patterns (Table 4, Figure 9). Vertical stratification of the water column due to hydrological properties presents barriers to mixing among different water masses [54]. Therefore, the bacterial communities in different water masses can be relatively isolated from each other [74] because of their relatively restricted mobility across such large scales. The community dynamics can then evolve differently within different water masses, as driven by the different physicochemical properties, and this scale of change will be dependent on factors including the duration of isolation and factors that influence the rate of growth (e.g., temperature, nutrients) and taxon-specific mortality. In this study, the overall trends for total bacterial diversity and community structure, as well as those for two major sub-groups of marine bacteria, the Rhodobacterales and Pelagibacterales, show how water mass plays a significant role in segregating communities. However, not all taxonomic groups follow the same patterns. The Rhodobacterales and Pelagibacterales groups, both from the Alphaproteobacteria, have distinct distribution patterns and responses to environmental factors.

### 4.1. The Mackenzie River Plume

River discharge has a strong impact on the physical properties of the surrounding seawater, as was found for our coastal site (Table 1). Not surprisingly, this greatly affected the bacterial communities at the coastal site and warrants discussion of this site separate from the offshore samples. There was also substantial variation at this site, as the 5-m sample has much lower salinity, indicating a stronger impact from the incoming freshwater, while the 35-m sample had a lower temperature and higher salinity. Distinctions between these depths are clearly borne out by analysis of the two samples’ physicochemical properties (Figure 2) and these differences are matched by the presence of distinct bacterial communities at the two depths (Figure 10). This fits with previous observations, such as the drastic changes in bacterial communities documented along an estuarine gradient [75] where many factors, including salinity, DOC and other nutrient availability, were found to be important. The riverine influence also resulted in distinct bacterial communities relative to the offshore sites (Figure 9 and Figure 10).

### 4.2. Water Mass Segregation and Bacterial Community Composition

The vertical water mass segregation at the Arctic Ocean offshore sites is a proxy of temperature, salinity, nutrient concentrations, and also light penetration, which depends on depth and ice coverage [54]. In this study, water mass is the most influential factor on the bacterial community composition in a given sample, with significant effects on bacterial diversity (Table 4) and community structure (Figure 9 and Figure 10). Water mass was described as the driving factor for bacterial communities across various basins in the deep Arctic and North Atlantic Oceans [17,76], and our findings show it also plays a critical role in the upper Arctic Ocean.

Vertical stratification in the water column has long been proposed as an important factor for microbial distributions. A time series study in southern California suggested that the bacterial community was homogenized during the winter, when vertical stratification is weak, but becomes vertically divergent when the surface water warmed up and stratified [77]. In our system, the vertical stratification is not only due to the temperature-salinity profile, which creates a mixing barrier, but is also complicated by the diverse origins of the water at different depths. Water from the Atlantic Ocean sinks below the fresher Pacific-source water when it enters the Arctic Ocean, and the surface water is mainly from freshwater river discharge and ice melt [54]. Therefore, at the offshore sites in our study, there are three distinct water masses through the depths sampled. The surface is defined as the upper polar mixed layer (UPML), mainly formed by river discharge and melting sea ice. Below this is the lower polar mixed layer (LPML), mainly of Arctic origin, and then the Pacific summer water (PSW) is our lowest sampled water mass. Fitting with the “barrier to dispersal” theory [74], these water masses of different origins would bring their source bacterial communities into the new location, where they would be subsequently shaped according to changes in the environmental conditions, but retain distinct signatures in the absence of homogenization with other water sources. This is also reflected in a previous study where Baffin Bay water was found to have similar bacterial communities as samples from the Canada Basin halocline in the Arctic Ocean [17], consistent with the movement of water from the Canada Basin into Baffin Bay.

### 4.3. Community Structure within Water Masses

Across the approximately 400-km distance separating the sampling stations in this study, there is no significant difference in the bacterial communities for samples collected from the same water mass. This can be explained, in part, by the circumpolar circulation via the Beaufort Gyre in the Arctic Ocean [54,78]. Although there are discrepancies about the proportions of water inputs from various sources, these studies generally agree that within the Canada Basin the primary circulation of water is counterclockwise. This circulation will distribute microbial communities within individual water masses horizontally throughout the Ocean in the absence of mixing, upwelling or sinking, and there is evidence that the Arctic Ocean microbial communities are very stable over time [39]. Even when the environmental conditions change drastically, such as summer to winter, most bacterial groups remain stable [39]. Therefore, we can also assume that the bacterial communities we characterized in this study would represent any other location from the same water masses well.

### 4.4. Dominance of Alphaproteobacteria in the Beaufort Sea

The class Alphaproteobacteria was by far the most abundant taxonomic group across our study samples, represented largely by the Rhodobacterales and Pelagibacterales orders. The Pelagibacterales comprised three OTUs and showed little variation among offshore samples, with respect to relative abundance or diversity. However, the Pelagibacterales grouping according to water mass is supported less (Figure 9 and Figure 10, Table 4).

The most abundant Pelagibacterales OTU, making up approximately 80% of the sequences from that group, is 100% identical to the Arctic 95B-1 sequence [63] and corresponds to subclade IIa according to the BATS SAR11 ecotypes [62]. The representative sequence Arctic 95B-1 was first described in the Arctic Ocean [63], and in Bermuda this ecotype annually reaches high abundance in the upper 200 m in the deep-mixing fall-winter season and is described as a winter surface ecotype [62]. The high proportion of this OTU in our Arctic Ocean samples supports this subclade’s description as a cold-water group, although the mechanism for its success in this environment is yet to be determined by investigating cultured strains. The second Pelagibacterales OTU made up approximately 13% of this group’s population and corresponds to subclade Ib, described as a winter-spring subclade in the BATS ecotypes [62]. At the BATS site, subclade Ib is most abundant in the deepest mixed-layer and is much more abundant at its peak compared to subclade IIa. It is possible that there are similar shifts among these different ecotypes in the Arctic Ocean, which we cannot directly address because our samples only represent a single time point. However, as mentioned already, communities in the Arctic Ocean have been documented to be comparatively stable over time [39]. The third OTU, at approximately 8% of the Pelagibacterales population and found in all our Arctic Ocean samples, does not closely match any of the subclade representatives (Figure 7) and could represent a novel undefined lineage.

The distribution of the Rhodobacterales is distinctly different from the Pelagibacterales. They were very abundant at the surface, exceeding Pelagibacterales at all stations except the furthest offshore (Figure 6). Whereas the Pelagibacterales are consistent across all stations and depths, the Rhodobacterales abundance decreased dramatically from the surface (UPML) to the deeper water mass (PSW). Within this group, several roseobacters that appear to be generally successful and abundant in northern oceans were also abundant in our communities. Sequences associated with the DC5–80-3 (also referred to as RCA), NAC11–7, and OCT groups were the most abundant. The DC5–80-3 and NAC11–7 sequences decreased with depth whereas the OCT group had the opposite trend and peeked in the deep offshore samples. The abundance of the DC5–80-3 group is the driver of the lower Rhodobacterales diversity in the surface samples.

Overall, the DC5–80-3 lineage was the most abundant, contributing up to 91% of the Rhodobacterales sequences in our samples. This lineage has been detected as highly abundant in surface waters in several previous studies [28,79,80], including in the Southern Ocean where it showed a seasonal response to changes in temperature, latitude, and nutrient concentrations [81]. It is active in temperate to subpolar oceans, with 93% of the genome transcribed in situ in the North Sea [79,82]. A representative strain from this lineage, *Planktomarina temperata* RCA23 [83], has been characterized and showed evidence of genome streamlining, with the smallest genome among roseobacters [83,84]. This feature, similar to what is found for Pelagibacterales [85,86], is believed to be an important contributor to its success in these oceanic environments and our study further documented its prevalence and distribution patterns in the Arctic Ocean.

The NAC11–7 group is also abundant in the Arctic Ocean, particularly in the Mackenzie River-affected site and offshore surface waters. This group is a prominent component in Southern Ocean bacterial communities [81,87] and is recognized for its ability to consume dimethylsulfoniopropionate (DMSP) [88,89], potential for photoheterotrophy [31] and being active in the community [87,90]. In our study, NAC11–7 made up 5% of the total community and 37% of the Rhodobacterales at the coastal site. Offshore, it decreased with depth overall, but its proportion within the Rhodobacterales increased from the surface to depth.

The last major Rhodobacterales group, OCT, is at its highest relative abundance within the Rhodobacterales in the deepest offshore samples. The differential patterns for these three major Rhodobacterales lineages support niche differentiation among them, with DC5–80-3 thriving at the surface, NAC11–7 most abundant at the coastal station, and OCT being predominant in deeper water.

### 4.5. Other Abundant Groups

Gammaproteobacteria was the second-most abundant higher-level taxon, reaching as high as 18% of the total community offshore and 32% at the coastal site. At the coastal site these bacteria increased in abundance with depth and were represented predominantly by the orders Alteromonadales and Thiotrichales. Offshore, their highest abundance was found at the intermediate depths (in LPML) where the Chl *a* concentration was highest. Overall, these patterns fit with previous associations of this group with phytoplankton and responses to increased Chl *a* and DOC [91,92], and as an abundant group in Arctic waters [39,93,94,95].

### 4.6. Influence of Environmental Factors on Bacterial Communities

The effects of environmental factors on the bacterial communities in our study are inconclusive. The strongest impacting factor is water mass, a proxy of temperature, salinity, and, to some extent, nutrients. When the water column is vertically stratified, water mass would also be affected by light availability. We were not able to delineate the specific contributions from each aspect of water mass on the overall bacterial community structure. Although water mass segregation is the most impacting factor on bacterial community structure and diversity, each taxonomic group responded to water mass differently. Among the two taxonomic groups that we analyzed in detail, Rhodobacterales had the most significant correlation with water mass, whereas Pelagibacterales showed a much less profound correlation (Figure 6, Table 4). This likely reflects the distinct adaptation strategies these two groups use for their successful oceanic existence. Members of the Rhodobacterales are characterized as having versatile metabolic capabilities [96] and being capable of responding to changing environmental factors quickly. In contrast, Pelagibacterales have extremely streamlined genomes that help maximize their efficiency through lower-cost maintenance and reproduction [97]. Although this might restrict their capacity to cope with rapidly changing environments, they exist as stable populations with a cosmopolitan existence and dominate the global oceans overall [37,79,81,94,98,99,100].

## 5. Conclusions

In this study, we have found that the Arctic Ocean bacterial community is mostly controlled by the vertical water mass segregation. Water mass distinction is largely based on temperature and salinity patterns, and there are clearly mixing barriers in this Arctic Ocean system that perpetuate community distinctions among these masses. Alphaproteobacteria dominated all samples, mostly comprising the Rhodobacterales and Pelagibacterales orders. The different bacterial lineages show distinct distribution patterns, but all reflect the impact of this water mass segregation. Polar oceans have unique physical, biological and biogeochemical characteristics and community structures, and the potential for climate and/or anthropogenic influences to impact bacterial communities in the Arctic Ocean is significant [101,102]. For example, these could alter water mass mixing and the picture presented here based on samples collected in 2009 will provide an important reference point for future studies searching for evidence of change in the Arctic Ocean.

## Figures and Tables

**Table 1:** Physical and hydrographical parameters for samples in this study.

Sample ID	Date	Longitude (°W)	Latitude (°N)	Depth (m)	Relative Light Intensity (%)	Water Mass	Temp. (°C)	Salinity (psu)	Sea Ice Coverage (%)
C-5	2009–08-30	137.9964	69.5008	4.7	50	Coastal	3.13	23.75	0
C-35	2009–08-30	137.9964	69.5008	35	1	Coastal	0.58	30.40	0
O1–10	2009–09-01	139.0255	71.1059	10	Surface	UPML	−1.02	25.78	70
O1–21	2009–08-31	139.0090	71.0922	21.8	50	UPML	−0.90	28.18	70
O1–85	2009–08-31	139.0090	71.0922	85	1	PSW	−1.27	31.75	70
O2–10	2009–09-09	136.7900	72.5158	9.9	50	UPML	−1.34	25.26	90
O2–70	2009–09-09	136.7900	72.5158	69.5	1	LPML	−1.06	30.67	90
O2–140	2009–09-09	136.7900	72.5158	139.8	0.1	PSW	−1.36	32.46	90
O3–2.5	2009–09-04	137.1223	74.5937	2.7	Surface	UPML	−1.41	26.71	90
O3–55	2009–09-04	137.1223	74.5937	54.6	1	LPML	−1.03	30.56	90
O3–100	2009–09-04	137.1223	74.5937	100	0.1	PSW	−1.16	31.98	90

**Table 2:** Bacterial properties and Chl *a* concentrations for samples in this study.

Sample ID	Bacterial Biomass (µg C L^−1^)	Bacterial Abundance (10^3^ cells mL^−1^)	Cellular Carbon (fg/cell)	Bacterial Production (µg C L^−1^ d^−1^)	Bacterial Growth Rate (d^−1^)	Chl *a* (µg L^−1^)
C-5	9.03	530	17.04	0.62	0.06	0.14
C-35	9.57	417	22.95	1.22	0.15	0.11
O1–10	3.63	200	17.99	0.11	0.03	0.18
O1–21	3.68	222	16.56	0.05	0.01	0.05
O1–85	2.08	124	16.81	0.03	0.01	0.12
O2–10	3.80	230	16.53	0.23	0.06	0.06
O2–70	3.65	281	13.00	0.09	0.02	0.15
O2–140	1.28	88	14.58	0.01	0.01	0.01
O3–2.5	3.99	237	16.85	0.14	0.03	0.09
O3–55	4.00	236	16.96	0.06	0.02	0.19
O3–100	1.90	117	16.22	0.02	0.01	0.03

**Table 3:** Community characteristics for total bacterial communities, the order Rhodobacterales and Pelagibacterales for each sample.

	Number of Sequences	Number of OTUs	Shannon Index (*H’*)	Chao	Inverse Simpson (1/*D*)
Total community					
C-5	181,488	3886	3.83	10,619.34	14.003
C-35	205,484	2805	3.37	7242.58	11.881
O1–10	158,302	4177	2.87	6544.76	5.668
O1–21	153,607	2333	3.18	5465.40	7.192
O1–85	185,815	2201	4.11	11,808.09	10.470
O2–10	150,701	4501	3.11	6861.16	6.048
O2–70	99,495	2334	3.97	6391.22	12.284
O2–140	162,668	2473	4.30	13,157.34	11.756
O3–2.5	48,356	1200	3.25	2781.98	7.199
O3–55	142,258	3444	4.19	9256.73	12.481
O3–100	125,773	3756	4.45	9982.94	13.938
Rhodobacterales					
C-5	44,137	461	1.62	3143.73	2.786
C-35	30,626	300	1.74	1860.32	3.968
O1–10	53,691	284	0.54	1718.79	1.220
O1–21	30,117	195	0.70	1295.25	1.340
O1–85	6598	196	2.31	728.83	4.630
O2–10	57,235	346	0.75	1692.90	1.420
O2–70	8586	127	1.35	564.10	2.278
O2–140	5346	222	2.57	1037.06	6.135
O3–2.5	12,362	106	0.72	448.33	1.370
O3–55	12,006	191	1.63	994.08	2.732
O3–100	5295	202	2.59	841.40	6.329
Pelagibacterales					
C-5	34,209	339	0.73	1340.28	1.399
C-35	26,576	211	0.65	1106.59	1.330
O1–10	43,824	323	0.93	1809.57	1.731
O1–21	54,449	313	0.86	1493.67	1.623
O1–85	50,809	415	0.84	1846.03	1.494
O2–10	36,343	269	0.97	1266.5	1.814
O2–70	26,518	222	0.79	914.652	1.514
O2–140	37,500	419	1.04	2394.55	1.704
O3–2.5	15,565	153	0.93	543.056	1.711
O3–55	38,762	352	0.80	1346.38	1.468
O3–100	28,721	285	1.00	1177.22	1.714

**Table 4:** Relationship between Shannon diversity (*H’*) and environmental factors.

Variable ^1^	Total Community		Rhodobacterales		Pelagibacterales	
	F	*p*-Value	F	*p*-value	F	*p*-Value
Water Mass	**23.93 ^2^**	**<0.001**	**109.52**	**<0.001**	**8.13**	**0.011**
Sampling station	0.51	0.686	0.19	0.900	3.10	0.099
	*r*	*p*-value	*r*	*p*-value	*r*	*p*-value
Temperature	−0.011	0.975	0.059	0.862	**−0.639**	**0.034**
Salinity	**0.703**	**0.016**	**0.736**	**0.010**	0.103	0.763
Density	**0.701**	**0.016**	**0.721**	**0.012**	0.139	0.684
BB	−0.316	0.344	−0.196	0.564	**−0.816**	**0.002**
BA	−0.267	0.427	−0.258	0.444	**−0.794**	**0.004**
BP	−0.258	0.444	−0.000	1.000	**−0.720**	**0.013**
BGR	−0.374	0.257	−0.112	0.744	**−0.624**	**0.040**
Chl *a*	−0.175	0.606	−0.336	0.312	−0.584	0.059

^1^ ANOVA used for water mass and sampling station; Pearson correlation used for temperature, salinity, density, bacterial biomass, bacterial abundance, bacterial production, bacterial growth rate, and Chl *a* concentration. ^2^ Bold indicates *p* < 0.05.

## Supplementary Materials

The following are available online at https://www.mdpi.com/2076-2607/7/10/385/s1, Table S1. Information for SAR11 reference sequences, Table S2. PCA correlation coordinates of all environmental parameters for analyses done at different taxonomic levels, Table S3. Inorganic nutrient concentrations for samples in our study, Figure S1. Principal component analysis (PCA) based on the physical and chemical properties of 10 samples, Figure S2. Relationship between sequencing effort and diversity metrics.
