# Supplementary material for: Effects of Vertical Water Mass Segregation on Bacterial Community Structure in the Beaufort Sea
Source: Microorganisms. 2019 Sep 24;7(10):385. doi: 10.3390/microorganisms7100385 (PMC6843845; doi:10.3390/microorganisms7100385)
Supplement: Supplementary file 1 [file microorganisms-07-00385-s001.pdf]

Table S1. Information for SAR11 reference sequences.

SAR11 subclade reference sequence	NCBI accession number	Subclade	Type of sequence	Reference[a]
Pelagibacter ubique HTCC1002	NZ_CH724130.1	Ia	genome	[64]
SAR193	U75649.1	Ib	clone	[6]
SAR11	X52172.1	Ib	clone	[22]
SAR211	U75256.1	IIa	clone	[6]
Arctic95B-1	AF353214.1	IIa	clone	[63]
SAR203	U75255.1	IIb	clone	[6]
OM155	U70686.1	IIIa	clone	[65]
HIMB114	ADAC02000001.1	IIIa	genome	[64]
HIMB59	NC_018644.1	Va	genome	[64]

[a] Reference numbers correspond to the reference list in the main manuscript

Table S2. PCA correlation coordinates of all environmental parameters for analyses done at different taxonomic levels.

Bacterial phyla				
	PC1	PC2	r	p-value
Station	-0.965	0.261	0.241	0.326
**Depth**[a]	**-0.975**	**-0.220**	**0.909**	**0.001**
**BB**	**0.883**	**-0.469**	**0.658**	**0.011**
**BA**	**0.846**	**-0.533**	**0.849**	**0.001**
BP	0.887	-0.461	0.267	0.270
BGR	0.982	-0.188	0.215	0.375
**Chl *a***	**0.554**	**-0.832**	**0.626**	**0.017**
**Water mass**	**-0.999**	**-0.032**	**0.846**	**0.001**
**Temperature**	**0.672**	**-0.740**	**0.630**	**0.014**
**Salinity**	**-0.987**	**-0.162**	**0.687**	**0.013**
Sea ice	-0.829	0.560	0.377	0.146
Proteobacteria				
	PC1	PC2	r	p-value
Station	0.999	-0.051	0.383	0.143
**Depth**	**0.853**	**-0.522**	**0.713**	**0.008**
**BB**	**-0.906**	**0.423**	**0.595**	**0.023**
**BA**	**-0.727**	**0.687**	**0.701**	**0.009**
BP	-0.997	-0.081	0.436	0.109
BGR	-0.981	-0.196	0.445	0.108
Chl *a*	0.197	0.980	0.469	0.086
**Water mass**	**0.930**	**-0.367**	**0.851**	**0.001**
Temperature	-0.761	0.649	0.563	0.060
**Salinity**	**0.816**	**-0.578**	**0.818**	**0.001**
Sea ice	0.984	-0.178	0.483	0.076
Rhodobacterales				
	PC1	PC2	r	p-value
Station	-0.988	0.156	0.339	0.172
**Depth**	**-0.691**	**-0.723**	**0.876**	**0.002**
**BB**	**1.000**	**0.019**	**0.608**	**0.020**
**BA**	**1.000**	**0.001**	**0.709**	**0.010**
BP	0.997	-0.083	0.331	0.167
BGR	0.975	0.223	0.248	0.254
Chl *a*	0.816	-0.578	0.095	0.695
**Water mass**	**-0.843**	**-0.538**	**0.843**	**0.003**
**Temperature**	**0.938**	**-0.346**	**0.895**	**0.008**
**Salinity**	**-0.837**	**-0.547**	**0.903**	**0.002**
**Sea ice**	**-0.965**	**0.263**	**0.600**	**0.016**
Pelagibacterales				
	PC1	PC2	r	p-value

Station	0.037	0.999	0.114	0.571
**Depth**	**-0.435**	**0.900**	**0.715**	**0.007**
BB	0.823	-0.568	0.378	0.151
BA	0.814	-0.581	0.412	0.109
BP	0.929	-0.369	0.270	0.288
BGR	0.916	-0.401	0.336	0.185
Chl *a*	0.817	0.577	0.058	0.790
**Water mass**	**-0.570**	**0.822**	**0.695**	**0.014**
Temperature	0.682	-0.732	0.205	0.415
**Salinity**	**-0.359**	**0.933**	**0.889**	**0.001**
Sea ice	-0.616	0.788	0.161	0.517

[a] Bold indicates parameter has significant correlation ($p < 0.05$) with the principal components

Table S3. Inorganic nutrient concentrations for samples in our study.

Sample ID	Nitrite (μM)	Nitrate (μM)	Silicate (μM)	Phosphate (μM)	Ammonium (μM)
C-5	0.17	0.16	5.80	0.43	0.01
C-35	0.20	0.30	4.35	0.71	0.04
O1-10	ND[a]	ND	ND	ND	ND
O1-21	0.09	0.04	1.85	0.66	0.03
O1-85	0.11	5.80	12.40	1.27	0.04
O2-10	0.13	0.00	1.98	0.49	0.02
O2-70	0.22	0.42	3.13	0.79	0.02
O2-140	0.12	14.64	30.23	1.89	0.03
O3-2.5	0.14	0.15	1.48	0.55	0.01
O3-55	0.18	0.46	2.66	0.80	0.03
O3-100	0.156	0.833	19.022	1.52	0.04

[a] ND, not determined.

Table S4. Number of sequences for each sample before and after the quality filtering process.

Sample ID	Number of raw reads	Number of reads after quality filtering
C-5	239,104	181,488
C-35	271,478	205,482
O1-10	213,756	158,302
O1-21	213,677	153,607
O1-85	260,739	185,815
O2-10	208,792	150,701
O2-70	137,564	99,495
O2-140	226,121	162,668
O3-2.5	66,751	48,356
O3-55	196,165	142,257
O3-100	179,282	125,773

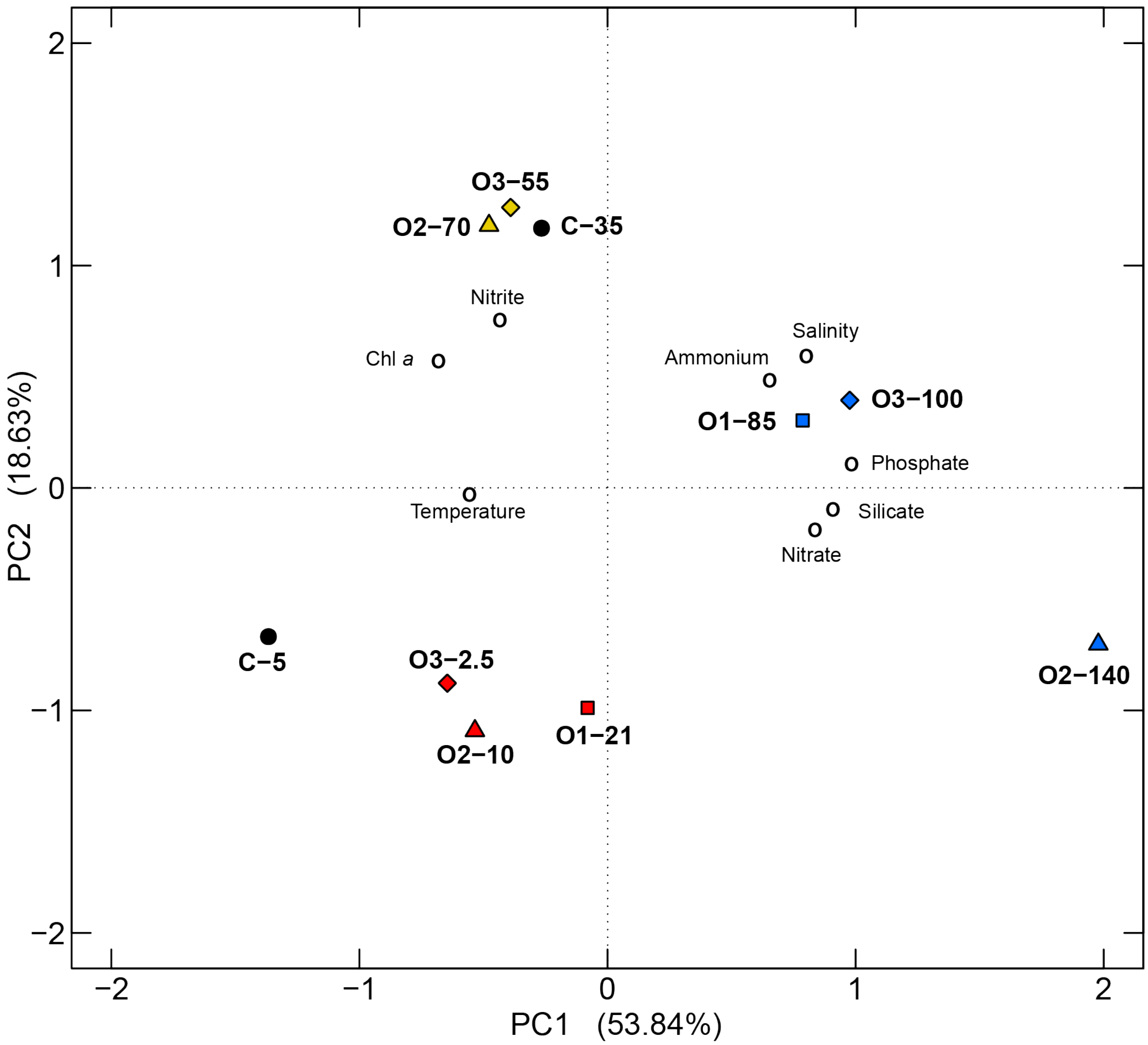

Figure S1. Principal component analysis (PCA) based on the physical and chemical properties of 10 samples. Sample O1-10 was not included due to lack of chemical data. The open circles represent the physical and chemical properties and the closed symbols represent the samples. The closed symbols are colored according to water mass: red, UPML; yellow, LPML; blue, PSW; black, coastal. The shapes represent different stations: C, circles; O1, squares; O2, triangles; O3, diamonds.

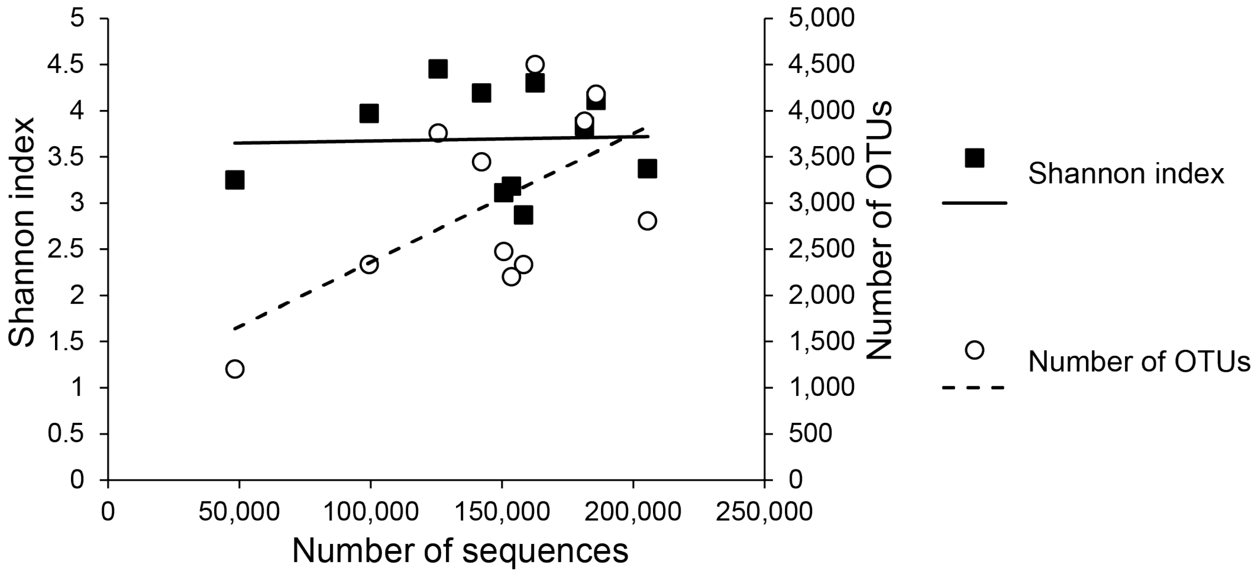

Figure S2. Relationship between sequencing effort and diversity metrics. The number of sequences included in the analyses for each sample are plotted against the number of OTUs (open circles) and the Shannon index (black squares).
